# Measuring prefrontal brain activity during verbal fluency tasks using functional near infrared spectroscopy

**DOI:** 10.3389/fneur.2026.1719660

**Published:** 2026-03-18

**Authors:** Nathan T. Palladino, Jia Anne Heng, Benjamin Wenzel, Kyla Kobylak, Ben Pyykkonen, William M. Struthers

**Affiliations:** Department of Psychology, Wheaton College, Chicago, IL, United States

**Keywords:** fNIRS, letter fluency, neuropsychology, semantic fluency, verbal fluency

## Abstract

**Introduction:**

Tasks of verbal fluency (VF) are common neuropsychological tests used for assessing executive function, language, and processing speed to identify impairments and aid in differential diagnosis. VF is usually divided into letter fluency (LF), quickly generating words beginning with a certain letter, and semantic fluency (SF), quickly naming words belonging to a category. Although both tasks utilize some executive function and language abilities, they have unique cognitive and neuroanatomical correlates, with LF traditionally being associated with more frontal and executive patterns while SF is seen as more temporal and linguistic. The current study aims to compare LF and SF cortical activation within the prefrontal cortex (PFC) in a non-clinical, English-speaking sample to explore how these VF tasks may both be relevant to frontal systems and how this can be applied clinically.

**Methods:**

This study uses functional near-infrared spectroscopy (fNIRS), a functional imaging method that utilizes near-infrared light to detect hemodynamic changes in cortical regions of the brain, to examine PFC activation during SF and LF tasks. Twenty-six English-speaking undergraduate participants performed 3 SF and 3 LF tasks. Differences in oxygenated and deoxygenated responses were compared between SF, LF, and individual baselines.

**Results:**

Results indicate that LF tasks required more PFC anterior resources and SF required more left posterior PFC resources, with both showing increased diffuse activation when compared to baseline.

**Discussion:**

These results (1) show activation of the PFC during SF and LF tasks and (2) have implications for clinical work, including potential for combining fNIRS with cognitive tests.

## 1 Introduction

Verbal fluency (VF) is a speeded, novel word-generation test, with individuals typically engaging in two similar but different verbal activities (1). Although other VF tasks exist, two primary versions are very common within neuropsychological evaluations; namely, letter fluency (LF; sometimes called phonemic fluency) and semantic fluency (SF). LF requires participants to quickly generate words beginning with a specific letter, and SF asks participants to name words belonging to a specified conceptual category, such as animals, furniture, or vegetables (2–4). The cognitive processes that are involved with both letter and semantic fluency include language abilities, attention, processing speed, and executive functions, such as self-monitoring and working memory (5). SF and LF are often used in conjunction with each other to aid in differential diagnosis, particularly for detecting impairment in semantic networks of the brain, such as in Alzheimer’s Disease (21). This is typically supported by the unique brain regions required in the different VF tasks.

Anatomical structures and their relationship to verbal fluency have been studied, but there have been controversies within the literature (5). A meta-analysis of individuals with focal cortical lesions (e.g., stroke, tumor, traumatic brain injury) found individuals with unilateral left frontal lesions had a larger negative impact on their performance for LF compared to healthy controls without neurological or psychiatric conditions (6). In a study with 1,231 individuals presenting to hospitals with acute ischemic stroke, Biesbroek et al. (5) found that SF is anatomically correlated with the temporal gyri and parts of the inferior frontal gyri. In contrast, they found that LF was more strongly associated with the anterior portions of the middle and inferior frontal gyri. Although there are notable differences in the neuroanatomical correlates between LF and SF, both types of VF were also associated with lesions to left frontotemporal and parietal cortical regions (5, 6). A magnetic resonance imaging (MRI) study comparing LF and SF showed lower performance on LF among individuals with frontal lesions and lower performance on SF among those with temporal lesions (22). A study of patients presenting with ischemic stroke found that participants with frontal damage had impaired performance on both LF and SF tasks (7). Taken all together, although SF has been shown to have temporal lobe associations, other evidence also points to the importance of the frontal lobe in both LF and SF.

Functional near infrared spectroscopy (fNIRS) is a method of analyzing brain activity by measuring changes in oxygenated and deoxygenated hemoglobin in the cortical areas of the brain. As a cortical region is activated, oxygenated hemoglobin increases and deoxygenated hemoglobin decreases. Assuming intact neurovascular coupling, these hemodynamic changes are indications of cortical activity, which fNIRS detects by sending near-infrared light through the scalp and measuring the returning light absorption, making it a helpful tool for identifying discrete cortical activation related to these VF tasks. Studies have reported a relationship between fNIRS and other neuroimaging techniques within the frontal lobe, including MRI (8), functional MRI (fMRI) (9), and electroencephalogram [EEG; (10)]. In contrast, fNIRS and positron emission tomography (PET) have not been widely studied because of the temporal differences between the two neuroimaging techniques (11).

Although fNIRS has been used to compare differences in brain activity during SF and LF tasks, these have not been done in English, with one study focusing on Chinese clinical populations (12) and the other on non-clinical populations with a Cantonese SF task (13). Although studies have used fNIRS in conjunction with a VF task to compare a clinical sample to healthy controls (14, 15), these did not compare activation of SF and LF. No current study has compared SF and LF in an English-speaking sample. The current study aims examine differences in LF and SF within the prefrontal cortex (PFC) in a non-clinical, English-speaking population to clarify the role of the PFC in VF tasks and begin to explore how this can be applied clinically.

## 2 Methods

Twenty-six undergraduate students aged 18–23 years old (*N* = 26; 22F/4M) from a Midwestern college in the United States were recruited for this study. Inclusion criteria included native, English-speaking individuals without fluency in another language and no neurological conditions. Participants were given a brief demographic survey, which included a self-report of languages spoken to ensure that inclusion criteria were met. They were then taken to a dimly lit, quiet testing room free from distractions, including no other people, noise, or activity.

**Figure 1:**
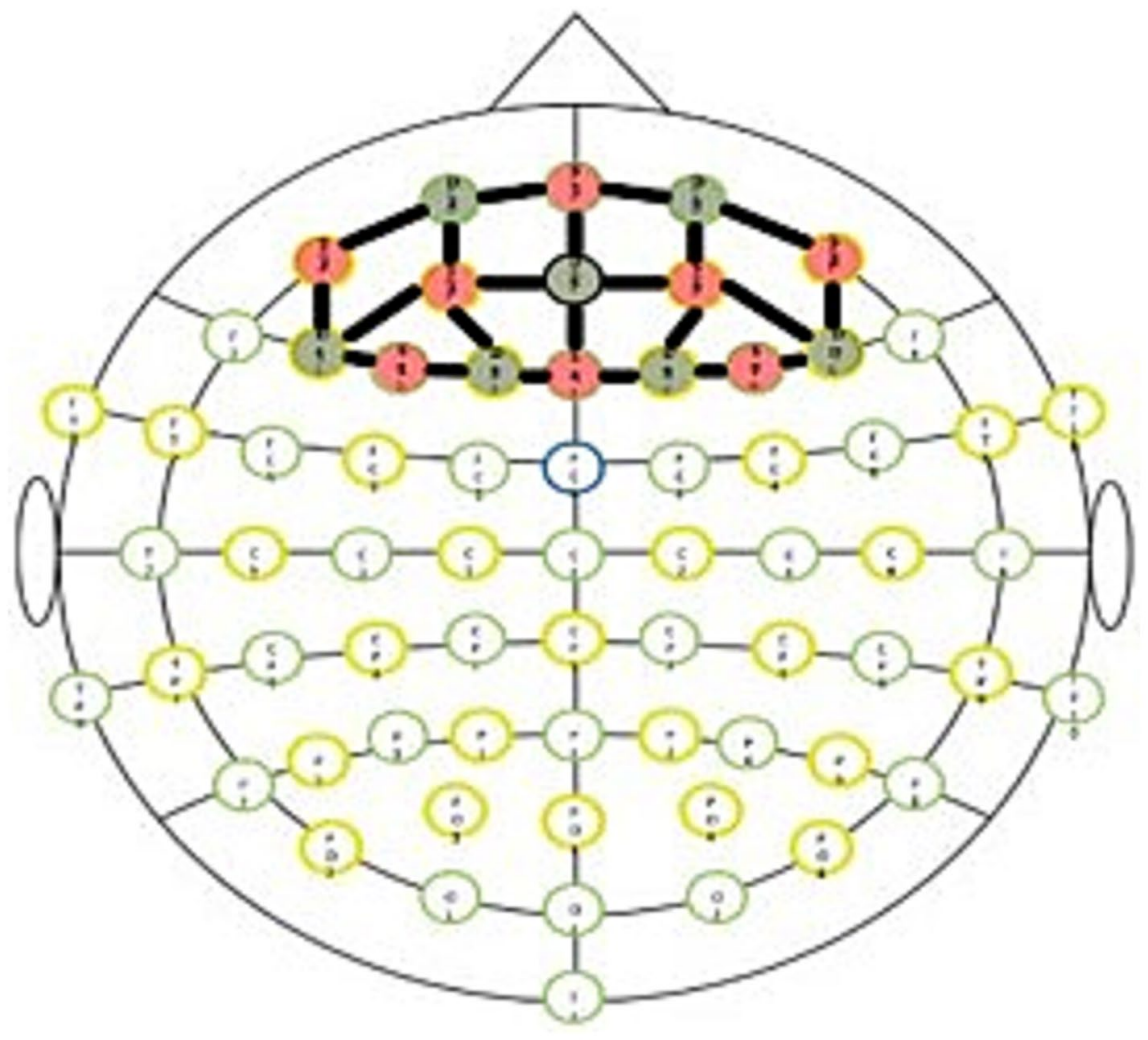
Prefrontal cortex montage.

Participants’ heads were manually measured and subsequently fitted with an 8 source x 7 detector NIRSport2 (version 2021.9.0) with short channel detectors fNIRS cap system (NIRx Medical Technologies). A prefrontal cortex (PFC) montage (see Figure 1) was utilized, which enabled 20 standard separation (~33 mm) cortical channels and seven short detector channels (~8 mm) to be measured. Optodes were placed according to the standard international 10–10 anatomical landmark system. The eight sources were placed at F3, AF7, AF3, Fz, Fpz, AF4, F4, and AF8 while the seven detectors were at F5, F1, Fp1, AFz, F2, Fp2, and F6. Our single device, NIRSport2 used dual wavelengths of 760 nm and 850 nm and a sampling rate of about 10.17 Hz.

Once fitted, all participants completed a randomized block design VF task using PsychoPy^1^ using a laptop computer. The block design included three 60-s timed letter prompts (F, A, S) and three 60-s timed semantic prompts (animals, fruit, professions). Prompt order was randomized across participants, with participants’ verbal responses for all prompts recorded and later scored. Two 30-s resting state baseline readings were taken, one before any tasks or instructions were given and another at the very end after a 20-s recovery period following the final task.

### 2.1 Data preprocessing

Data were run through a preprocessing workflow using Satori, an analytical fNIRS software part of NIRx Medical Technologies (16). Raw data were converted to optical density data before removing outliers using monotonic interpolation, rejecting channels using the scalp coupling index (SCI; threshold of 0.75), and correcting motion artifacts with temporal derivative distribution repair (TDDR). After converting to concentrated data, data were trimmed and z-transformed for normalization.

### 2.2 Statistical analyses

We used a multi-study general linear model (GLM) with predictors created from a convolution of a condition box-car time course and canonical two-gamma hemodynamic response function (HRF). Our GLM included three contrast blocks. First we compared the total oxygenated and deoxygenated hemoglobin between a resting state and the six blocks of VF. Given high amounts of data and risk of multiple comparisons, adjusting approach to significance testing is important to control for type I error (23). In this study, statistical significance will be determined using an uncorrected *p*-value of less than 0.001, consistent with other approaches in functional imaging (24–26). We also included a contrast in the GLM between the three blocks of SF and the three blocks of LF. These analyses were conducted using Satori, a neuroimaging software designed to analyze and visualize fNIRS data. More information on their built-in multi-study GLM feature is available in their user guide^2^ (16). Finally, a paired sample t-test examined possible differences in VF performance between the three trials of LF and the three trials of SF.

## 3 Results

### 3.1 VF results

Paired sample t-test (without assuming variance) of VF performance between the average number of words generated during LF (per trial Mean = 13.54, SD = 3.49; three trials combined Mean = 39.38, SD = 9.35) compared to SF (per trial Mean = 14.17, SD = 3.15; three trials combined Mean = 42.38, SD = 9.30) revealed no significant difference (*p* = 0.42). The two tasks were not meaningfully different in terms of cognitive performance.

### 3.2 VF compared to baseline results

Comparing the contrast of the total change in oxygenated hemoglobin (ΔHbO) and deoxygenated hemoglobin (ΔHbR) channels during a resting state to the three blocks of LF and three blocks of SF revealed a significant increase (*p* < 0.001) in cortical channels (*N* = 20) within the PFC in 20 LF ΔHbO channels and 18 SF ΔHbO channels, as well as a significant decrease in 19 LF ΔHbR channels and 19 SF ΔHbR channels (see Table 1). For LF, all channels except S1-D2 showed a significant decrease of ΔHbR compared to baseline, indicating a diffuse increase in activity. This is consistent with ΔHbO results, where each channel showed an increase in ΔHbO, indicating more activity during LF. SF similarly had diffuse decrease in ΔHbR compared to baseline, with the exception of S1-D2. When compared to baseline, SF’s ΔHbO was more variable, with four channels revealing higher ΔHbO during baseline, S1-D1, S3-D1, S6-D4, and S7-D5.

**Table 1:** Total ΔHbO and ΔHbR contrast of letter fluency (LF) compared to baseline, semantic fluency (SF) compared to baseline, and LF compared to SF.

Channel	LF vs. Baseline HbO		LF vs. Baseline HbR		SF vs. Baseline HbO		SF vs. Baseline HbR		LF vs. SF HbO		LF vs. SF HbR	
	*T*-Value	*p*-value	*T*-value	*p*-value	*T*-value	*p*-value	*T*-value	*p*-value	*T*-value	*p*-value	*T*-value	*p*-value
S1-D1	15.22	2.96E-52*	−19.06	5.74E-81*	−12.14	6.7E-34*	−23.73	2.4E-124*	30.58	5.03E-205*	5.22	1.80E-07*
S1-D2	24.39	2.9E-131*	18.32	6.71E-75*	8.56	1.12E-17*	20.61	2.91E-94*	17.70	4.49E-70*	−2.56	0.01
S2-D1	7.67	1.74E-14*	−47.07	0.00*	0.73	0.46	−35.74	6.4E-279*	7.75	8.95E-15*	−12.67	9.21E-37*
S2-D3	24.52	1.4E-132*	−50.78	0.00*	17.34	2.59E-67*	−12.35	4.96E-35*	8.03	1.02E-15*	−42.97	0.00*
S3-D1	32.49	5.6E-231*	−40.95	0.00*	−4.35	1.34E-05*	−47.36	0.00*	41.19	0.00*	7.16	7.84E-13*
S3-D3	32.08	2.3E-225*	−59.24	0.00*	30.98	2.5E-210*	−31.25	6.9E-214*	1.23	0.22	−31.30	1.20E-214*
S3-D4	26.03	4.1E-149*	−40.12	0.00*	6.26	3.91E-10*	−43.88	0.00*	22.10	3.90E-108*	4.20	2.71E-05*
S4-D2	16.40	2.13E-60*	−2.30	0.02	28.47	4.9E-178*	−9.56	1.21E-21*	−13.50	1.56E-41*	8.12	4.74E-16*
S4-D4	15.85	1.54E-56*	−23.80	4.8E-125*	8.78	1.58E-18*	−32.98	6.2E-238*	7.90	2.85E-15*	10.26	1.06E-24*
S4-D5	14.46	2.18E-47*	−15.38	2.39E-53	13.55	8.22E-42*	−34.90	4.3E-266*	1.02	0.31	21.82	1.90E-105*
S5-D3	14.29	2.49E-46*	−51.38	0.00*	2.48	0.01	−44.27	0.00*	13.21	7.80E-40*	−7.71	1.29E-14*
S5-D4	20.62	2.37E-94*	−43.27	0.00*	4.68	2.85E-06*	−33.87	7.9E-251*	17.82	5.85E-71*	−10.51	7.76E-26*
S5-D6	21.26	3.3E-100*	−39.33	0.00*	28.19	1.5E-174*	−10.21	1.84E-24*	−7.75	9.40E-15*	−32.56	4.70E-232*
S6-D4	18.88	1.92E-79*	−37.28	2.7E-303*	−4.24	2.24E-05*	−45.12	0.00*	25.85	4.00E-147*	8.76	1.96E-18*
S6-D6	19.37	1.65E-83*	−44.34	0.00*	26.92	2.4E-159*	−16.86	9.17E-64*	−8.44	3.16E-17*	−30.72	7.00E-207*
S6-D7	12.90	4.59E-38*	−45.85	0.00*	8.01	1.15E-15*	−43.49	0.00*	5.47	4.56E-08*	−2.63	0.008
S7-D5	9.40	5.72E-21*	−30.65	6.6E-206*	−3.74	1.86E-04*	−41.19	0.00*	14.68	8.47E-49*	11.79	4.65E-32*
S7-D7	23.52	3.2E-122*	−53.38	0.00*	22.40	4.8E-111*	−35.40	8.1E-274*	1.25	0.21	−20.10	8.16E-90*
S8-D6	35.89	3.1E-281*	−43.80	0.00*	36.60	1.9E-292*	−14.46	1.1E-168*	−0.80	0.42	−32.81	1.50E-235*
S8-D7	11.38	5.63E-30*	−51.59	0.00*	22.73	2.8E-114*	−27.71	0.24	−12.70	6.19E-37***	−26.71	6.50E-157*

Positive *T*-values indicate more ΔHbO or ΔHbR in the first item of each comparison pair, and a negative *T*-value indicates more ΔHbO or ΔHbR in the second item for each comparison pair. An increase in ΔHbO corresponds with more activity and an increase in ΔHbR corresponds with less activity. E.g., In LF vs. Baseline HbO, a positive *T*-value means more ΔHbO during LF, and a negative *T*-value means more ΔHbO during Baseline. *p < 0.001.

### 3.3 LF compared to SF results

**Figure 2:**
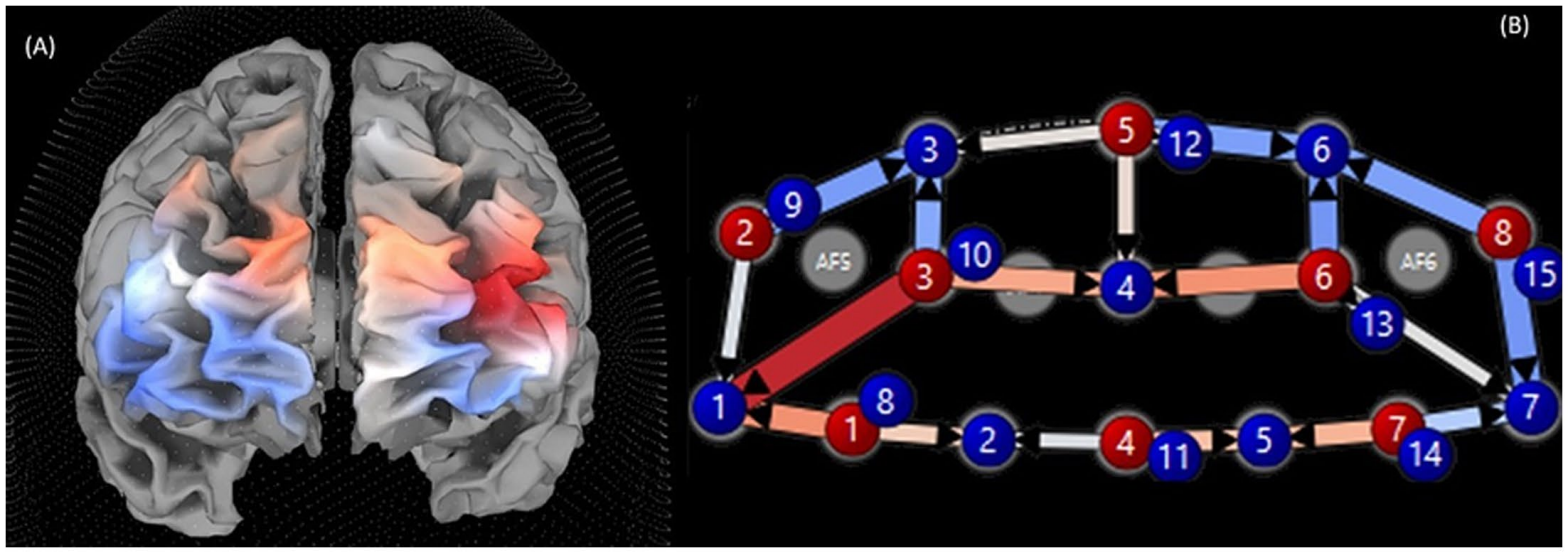
Visualizations of total hemoglobin (HbT) during letter fluency (LF) compared to semantic fluency (SF), where red represents SF tasks, and blue represents LF both views. **(A)** A 3D view constructed to show approximate regions of HbT in each fluency task. **(B)** A 2D view of HbT with channel numbers, where blue circles represent detectors and red circles represent sources.

A second contrast focused on differences in activity across the same 20 channels between LF and SF trial blocks (see Table 1 and Figure 2). Results showed significant differences (*p* < 0.001) in 18 of 20 channels. Of these 18 channels, SF’s ΔHbR was lower in 8 channels, concentrated more heavily in the left posterior PFC. Ten channels showed a lower ΔHbR for LF, primarily in the anterior PFC and right PFC. Alternatively, in the ΔHbO contrast, 16 of 20 channels were significantly different (*p* < 0.001). Eleven of these channels displayed higher ΔHbO during LF (generally left PFC) and five during SF (generally right anterior).

Beta values revealed that LF’s largest effect sizes (see Table 2) were the anterior PFC and left posterior PFC. Beta values during SF revealed that the largest effect sizes were primarily right posterior and medial posterior (see Table 2).

**Table 2:** Channel results of letter fluency (LF) and semantic fluency (SF), where beta values represent the strength and direction of hemodynamic response.

LF	HbO			HbR		
Channel	Beta value	*T*-value	*p*-value	Beta value	*T*-value	*p*-value
S1-D1	0.36	31.20	3E-213*	−0.08	−7.03	2.14E-12*
S1-D2	0.30	26.00	7.4E-149*	0.01	0.49	0.63
S2-D1	0.01	0.65	0.52	−0.51	−45.08	0.00*
S2-D3	0.17	14.80	1.45E-49*	−0.63	−55.13	0.00*
S3-D1	0.51	44.83	0.00*	−0.26	−22.30	4.9E-110*
S3-D3	0.16	13.81	2.27E-43*	−0.71	−62.55	0.00*
S3-D4	0.40	35.00	1.2E-267	−0.23	−20.26	3.6E-91*
S4-D2	0.12	10.04	1.08E-23	−0.03	−2.51	0.01
S4-D4	0.13	11.02	3.21E-28*	−0.18	−15.38	2.27E-53*
S4-D5	0.15	12.79	1.93E-37	−0.01	−1.01	0.31
S5-D3	0.23	19.62	1.13E-85*	−0.39	−34.53	1.4E-260*
S5-D4	0.26	22.29	5.7E-110*	−0.40	−34.58	2E-261*
S5-D6	0.09	7.92	2.3E-15*	−0.46	−40.43	0.00*
S6-D6	−0.003	−0.26	0.80	−0.56	−48.73	0.00*
S7-D5	0.15	12.82	1.34E-37*	−0.20	−17.30	4.83E-67*
S7-D7	0.18	15.67	2.59E-55*	−0.58	−50.43	0.00*
S8-D6	0.14	12.46	1.24E-35*	−0.49	−42.49	0.00*
S8-D7	−0.14	−12.37	3.77E-35*	−0.55	−48.04	0.00*

SF	HbO			HbR		
Channel	Beta value	*T*-value	*p*-value	Beta value	*T*-value	*p*-value
S1-D1	−0.13	−11.02	3.03E-28*	−0.16	−14.23	6.26E-46*
S1-D2	0.02	1.57	0.12	0.05	4.02	4.84E-05
S2-D1	−0.12	−10.05	8.97E-24*	−0.31	−27.59	2.80E-167*
S2-D3	0.04	3.73	19.5E-05*	0.05	4.18	2.85E-05*
S3-D1	−0.14	−12.03	2.53E-33*	−0.37	−32.19	8.10E-227*
S3-D3	0.14	12.11	9.55E-34*	−0.22	−19.34	2.85E-83*
S3-D4	0.05	4.49	7.29E-06	−0.30	−26.05	2.1E-149
S4-D2	0.33	28.68	1.6E-180	−0.16	−13.72	8.58E-43
S4-D4	0.001	0.11	0.91	−0.34	−29.55	1.60E-191*
S4-D5	0.13	11.38	5.35E-30	−0.36	−31.13	2.6E-212
S5-D3	0.02	1.39	0.17	−0.27	−23.89	6.00E-126*
S5-D4	−0.03	−2.30	0.02	−0.23	−20.07	1.50E-89*
S5-D6	0.21	18.62	2.52E-77*	0.05	4.52	6.23E-06*
S6-D6	0.13	11.40	4.45E-30*	−0.07	−6.32	2.67E-10*
S7-D5	−0.09	−7.45	9.08E-14*	−0.39	−33.58	1.50E-246*
S7-D7	0.16	13.94	3.85E-44*	−0.26	−22.67	1.10E-113*
S8-D6	0.16	13.57	6.54E-42*	−0.03	2.80	0.01
S8-D7	0.06	5.16	2.5E-07*	−0.13	−11.17	5.69E-29*

*p < 0.001.

## 4 Discussion

This study provides significant insights into the differential engagement of prefrontal regions during various VF tasks. The PFC is implicated in executive functioning skills including working memory, processing speed, organization, inhibition, and cognitive flexibility. The cognitive demands of VF are evidenced by the broad activation of the PFC observed in this study. Additionally, task-dependent differences between PFC activation during LF and SF tasks indicate distinct cognitive demands for each task.

Our findings demonstrate that the PFC is broadly activated by the VF tasks, compared to resting baseline activity. This observation reinforces the role of the PFC in VF, which is expected given the executive functioning required for VF tasks. Although both VF tasks showed diffuse activation relative to baseline, specific frontal regions were identified to relate to each VF task. Comparatively, LF was more associated with the anterior PFC while SF was associated with the posterior PFC. The anterior–posterior differences are consistent with findings on functional specialization of anterior and posterior areas in the PFC (27).

These findings are also consistent with previous studies that identified differential brain activation between LF and SF tasks. Zhu and Han (17) noted that tasks involving greater executive effort selectively involved regions in the anterior prefrontal lobe, and trials involving phonemic effort showed activation of the anterior PFC in the present study. The increased activation in LF may be attributed to the increased executive function effort required during LF tasks compared to SF, especially given that there was not a significant difference in the number of words generated between LF and SF in this study. Performances during LF tasks contrast with SF tasks as the latter is associated with posterior activation of the frontal lobe (18, 19).

Clinically, these results are noteworthy as poor performance on SF tasks cannot be solely attributed to temporal lobe dysfunction. Even in the context of high LF but low SF scores, our findings suggest that frontal systems could still be involved. Although VF tasks are helpful for differential diagnosis in conditions such as Alzheimer’s Disease (21), it may be suggested that SF could remain intact in the early stages of the disease, given the relative preservation of frontal areas in the initial stages of typical Alzheimer’s Disease (20, 28). Furthermore, in clinical settings, fNIRS has potential applications in conjunction with cognitive testing for patients being evaluated for frontal lobe dysfunction (e.g., attention-deficit/hyperactivity disorder), providing greater specificity in differential diagnosis and in conceptualizing overall cortical-level functioning.

### 4.1 Future directions

The external validity of this study is limited due to the size and homogeneity of the sample. Future fNIRS studies that focus on the relationship between activation of the PFC and VF tasks should consider a larger and more heterogeneous sample. In particular, the sample had primarily women, and future studies should aim to include a more even distribution of genders. There is a current ongoing study investigating a broader number of cortical regions involved in VF; specifically, we are looking to incorporate imaging of the temporal lobe with the PFC during VF, given its association with language abilities. Another limitation was that optodes were placed according to anatomical landmarks, rather than electronic 3-D spatial registration/positioning, which can reduce specificity and consistency of which brain regions are examined across participants.

### 4.2 Conclusion

Tasks of VF are typically associated with frontal and temporal regions of the brain. This is supported through both cognitive correlates of executive function and linguistic demand along with various techniques of brain imaging and lesion studies. The current study adds to this body of research through examining LF and SF using a fNIRS, a relatively cheaper and more portable method of measuring brain activity compared to others, such as fMRI. Results are consistent with existing conceptualizations of VF’s PFC correlates; namely, LF appeared more anterior while SF was more posterior. These findings also point to the potential utility of fNIRS in clinical settings, when used in conjunction with cognitive tests, to increase the specificity of interpreting patients’ performance.

## Data Availability

The raw data supporting the conclusions of this article will be made available by the authors, without undue reservation.

## Footnotes

1
https://www.psychopy.org

2
https://nirx.net/satori
